# Supplementary material for: Taf2 mediates DNA binding of Taf14
Source: Nat Commun. 2022 Jun 8;13:3177. doi: 10.1038/s41467-022-30937-w (PMC9177701; doi:10.1038/s41467-022-30937-w)
Supplement: Supplementary file 1 — Supplementary Information [file 41467_2022_30937_MOESM1_ESM.pdf]

Supplementary Information

Taf2 mediates DNA binding of Taf14

Brianna J. Klein, et al.

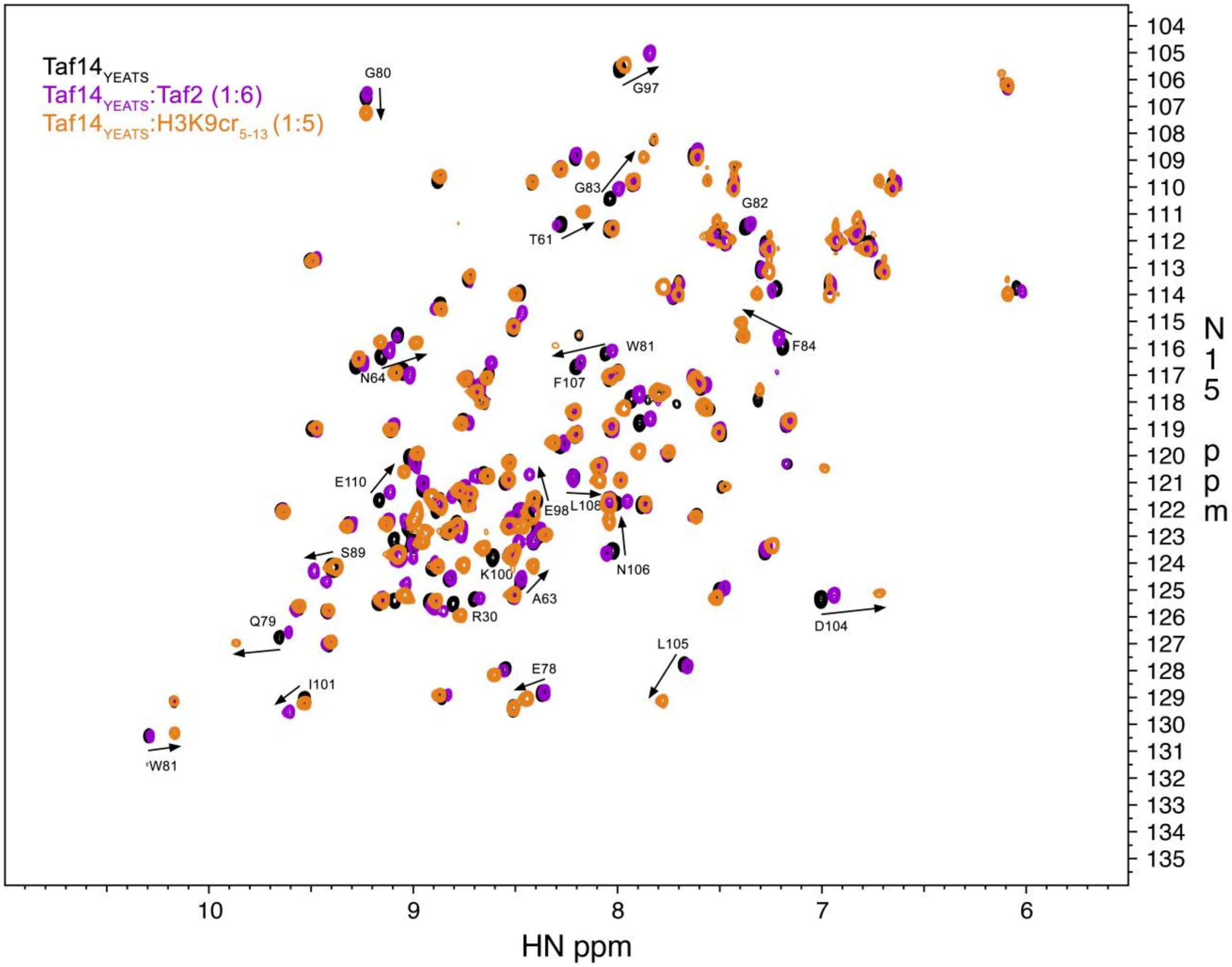

Supplementary Figure 1. Overlay of ^{1}H,^{15}N HSQC spectra of Taf14$_{YEATS}$ in the apo state (black) and bound to Taf2$_{CT}$ (purple) or H3K9cr$_{5\text{-}13}$ (orange). Related to Figure 2.

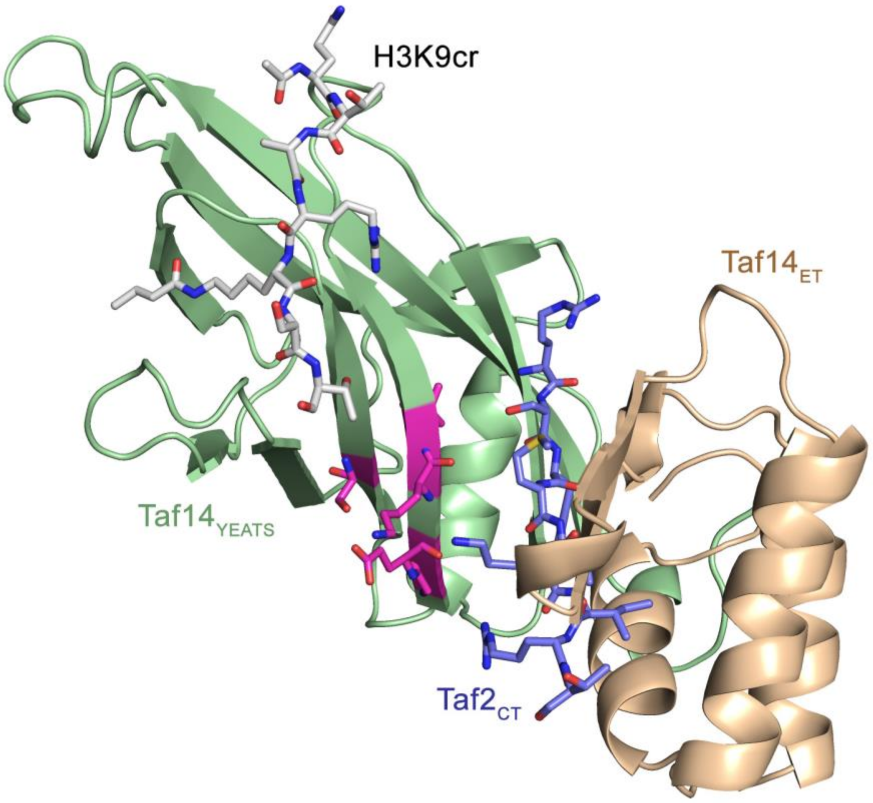

Supplementary Figure 2. Orientation of the crystal structures of the $Taf14_{YEATS}$:H3K9cr complex (light green:grey) and the $Taf14_{ET}$:$Taf2_{CT}$ complex (wheat:purple), as depicted in Figure 2. The residues of the $Taf2_{CT}$-binding site of $Taf14_{YEATS}$ are labeled and colored magenta. Related to Figure 2.

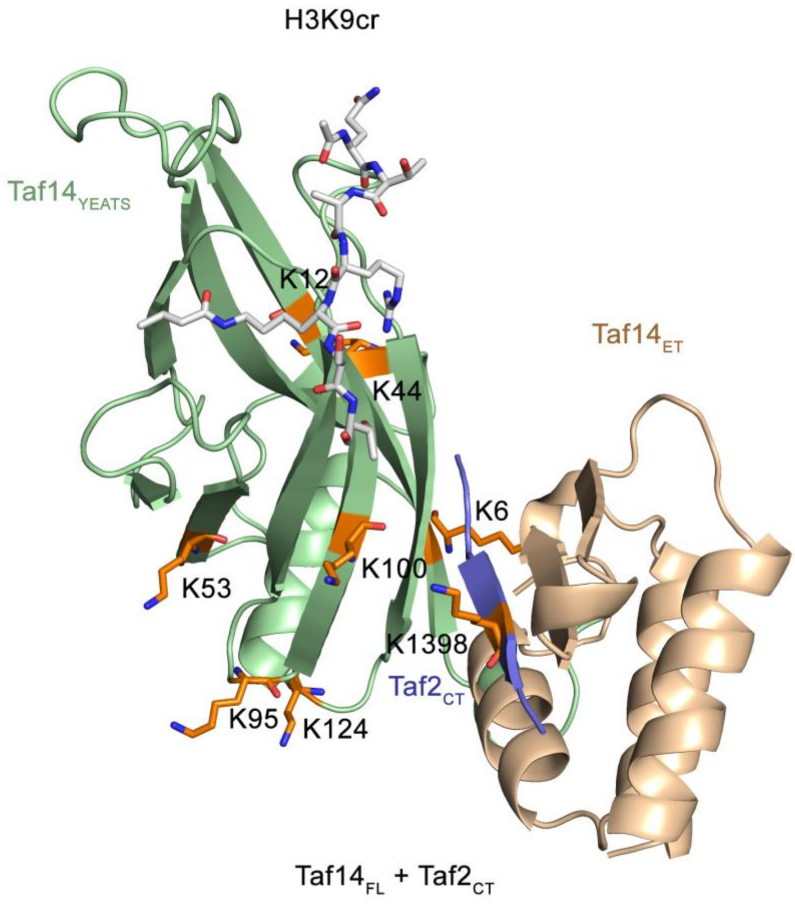

Supplementary Figure 3. Mass spectrometry analysis of the $Taf14_{FL}$:$Taf2_{CT}$ complex. The cross-link sites shown in Figure 2h are mapped on the structures of the $Taf14_{YEATS}$:H3K9cr complex (light green:grey) and the $Taf14_{ET}$:$Taf2_{CT}$ complex (wheat:purple). Crosslinked lysine residues are shown as orange sticks. Related to Figure 2.

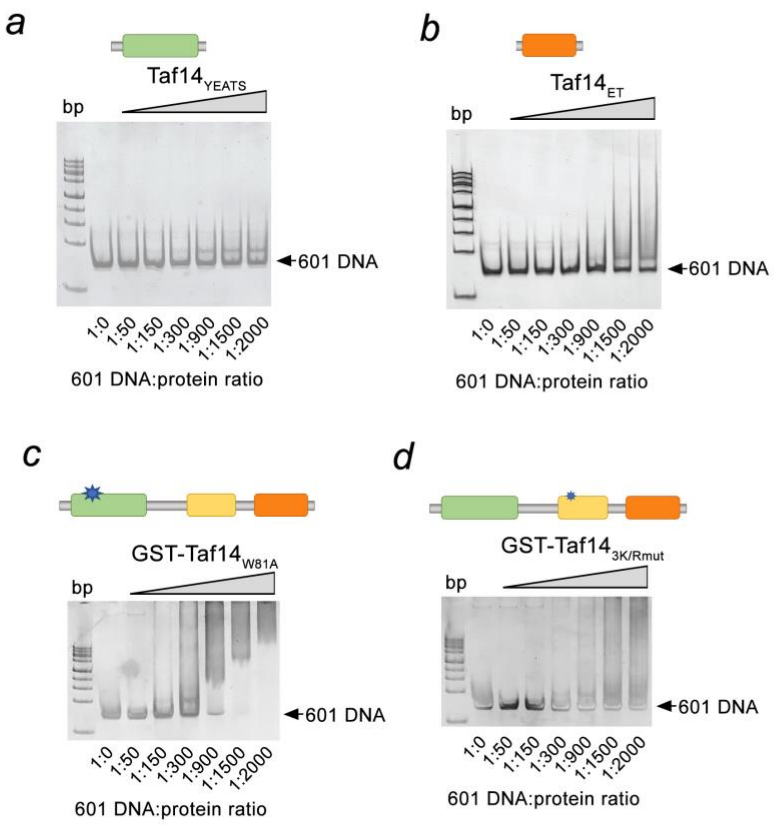

Supplementary Figure 4. Taf14 binding to 601 DNA. (a-d) EMSAs of 147 bp 601 DNA in the presence of increasing amounts of indicated Taf14 proteins. Architecture of each Taf14 construct is depicted above the gels and mutations are indicated with a blue star. Images are from single experiment. Source data are provided in a Source Data file. Related to Figure 3.

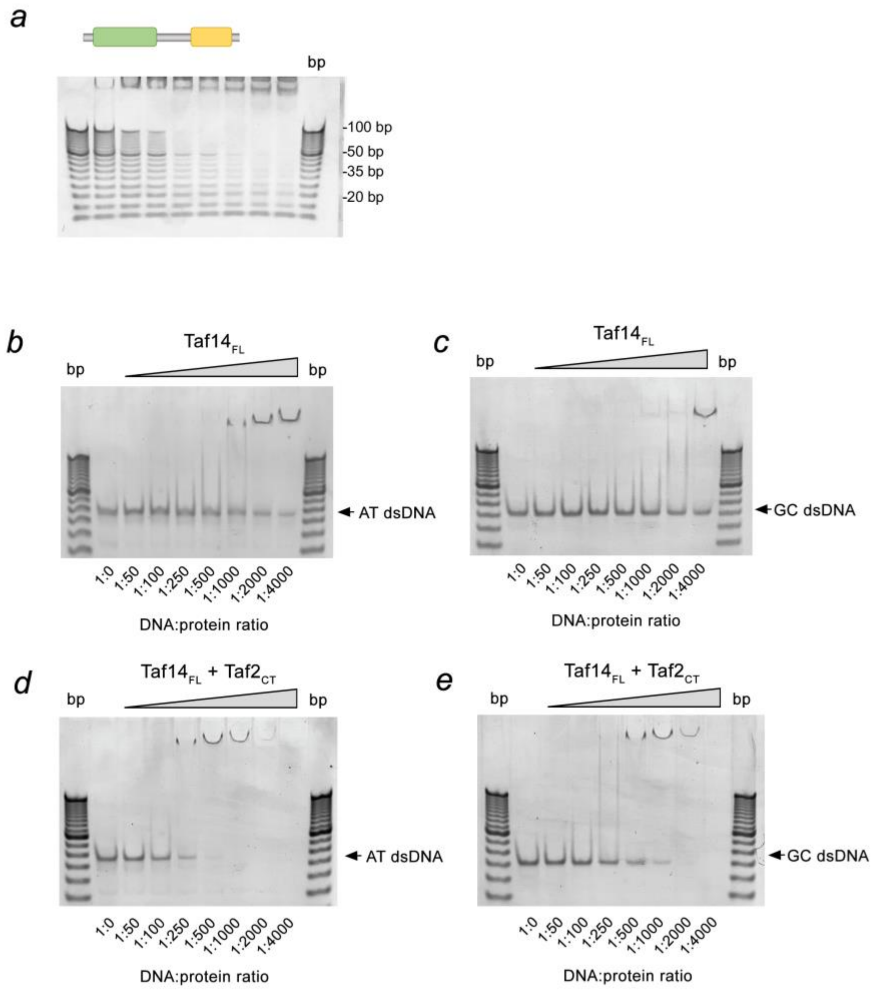

Supplementary Figure 5. Preference of Taf14 for DNA sequences evaluated by EMSA. (a) EMSA of a 5 bp dsDNA ladder in the presence of increasing amounts of GST-TAF14$_{\Delta ET}$, showing the minimum size of DNA necessary for the interaction. From left to right, the amount of Taf14 mixed with 25 µg/ml of ladder was: 12.5, 37.5, 75, 225, 300, 600, 900, and 1200 µM. (b, c) EMSAs of 36 bp AT rich (left) or GC rich (right) dsDNA in the presence of increasing amounts of Taf14$_{FL.}$ (d, e) EMSAs of 36 bp AT rich (left) or GC rich (right) dsDNA in the presence of increasing amounts of the Taf14$_{FL}$ + Taf2$_{CT}$ mixture. Taf14$_{FL}$ and Taf2$_{CT}$ were premixed at a 1:2 ratio prior incubation with DNA. The bp marker in (b-e) as in (a). Images are from single experiment. Source data are provided in a Source Data file. Related to Figure 3.

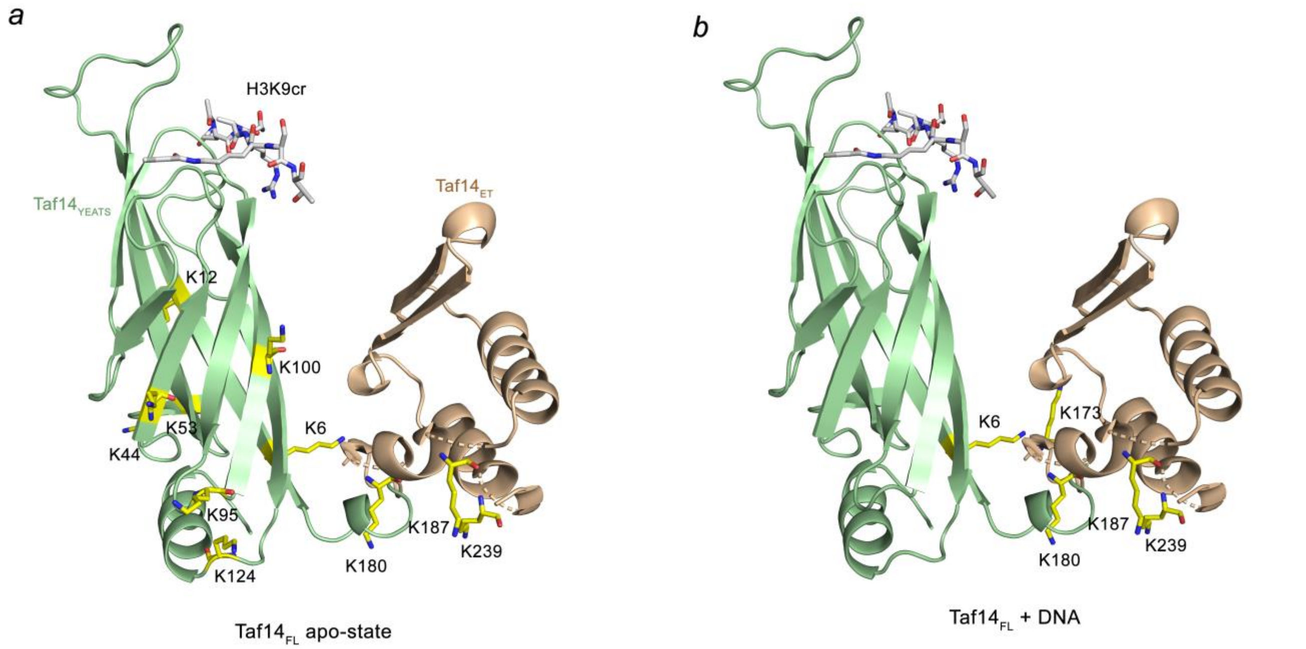

Supplementary Figure 6. Mass spectrometry analysis of the $Taf14_{FL}$ in the apo-state (a) and bound to DNA (b). The cross-link sites shown in Figures 3i and 3j are mapped on the structures of the $Taf14_{YEATS}$:H3K9cr complex (light green:grey) and the $Taf14_{ET}$:$Taf2_{CT}$ complex (only $Taf14_{ET}$ is depicted in wheat). Crosslinked lysine residues are shown as yellow sticks. Related to Figure 3.

a

Sample	Rg Guinier (Å)	Rg in reciprocal space (Å)	Rg in Real space (Å)	Dmax (Å)	I(0) Guinier	I(0) From GNOM	MW(KDa) Bayesian interference/ Credibility interval
Taf14	34.79±0.99	35.29	35.50±1.14	135	0.039±0.001	0.0385±0.0008	22.4 [19.6,29.3]
Taf14 withTaf2 Peptide	35.22±0.88	35.63	35.87±1.14	130	0.040±0.001	0.0396±0.0009	27.6 [25.3,29.3]
Taf14 with DNA	37.34±1.30	38.09	38.36±0.91	132	0.097±0.002	0.095±0.002	28.9 [27.9,33.5]

Rg, I(0) Guinier: Produced from Guinier plot
Rg , I(0) in Real space and Reciprocal space : Produced from GNOM

b

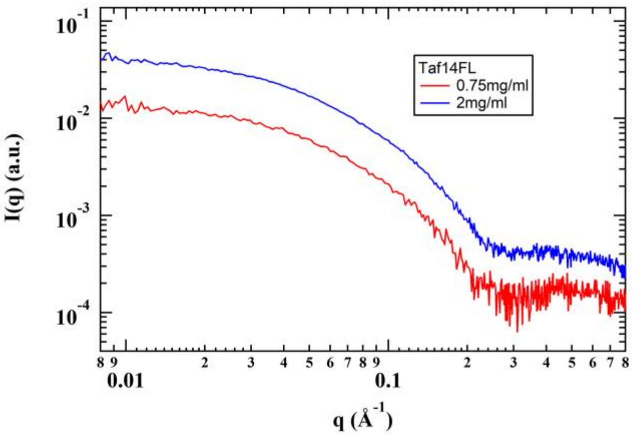

c

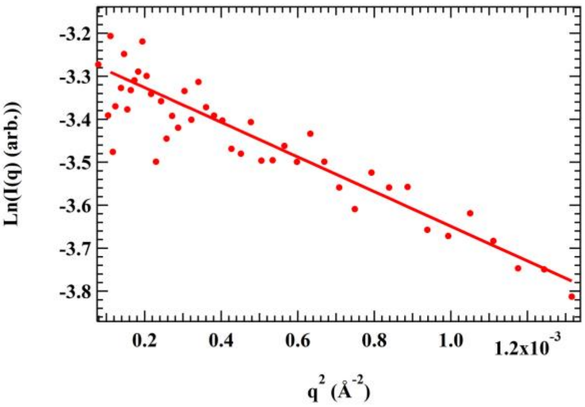

Supplementary Figure 7. (a) Integrated parameters obtained for the indicated samples from SAXS data. (b) SAX profiles at function of concentrations for Taf14$_{FL}$ in the apo- state. (c) Guinier plot of SAXS data extrapolated to zero concentration for Taf14$_{FL}$ in the apo- state. Related to Figure 3.

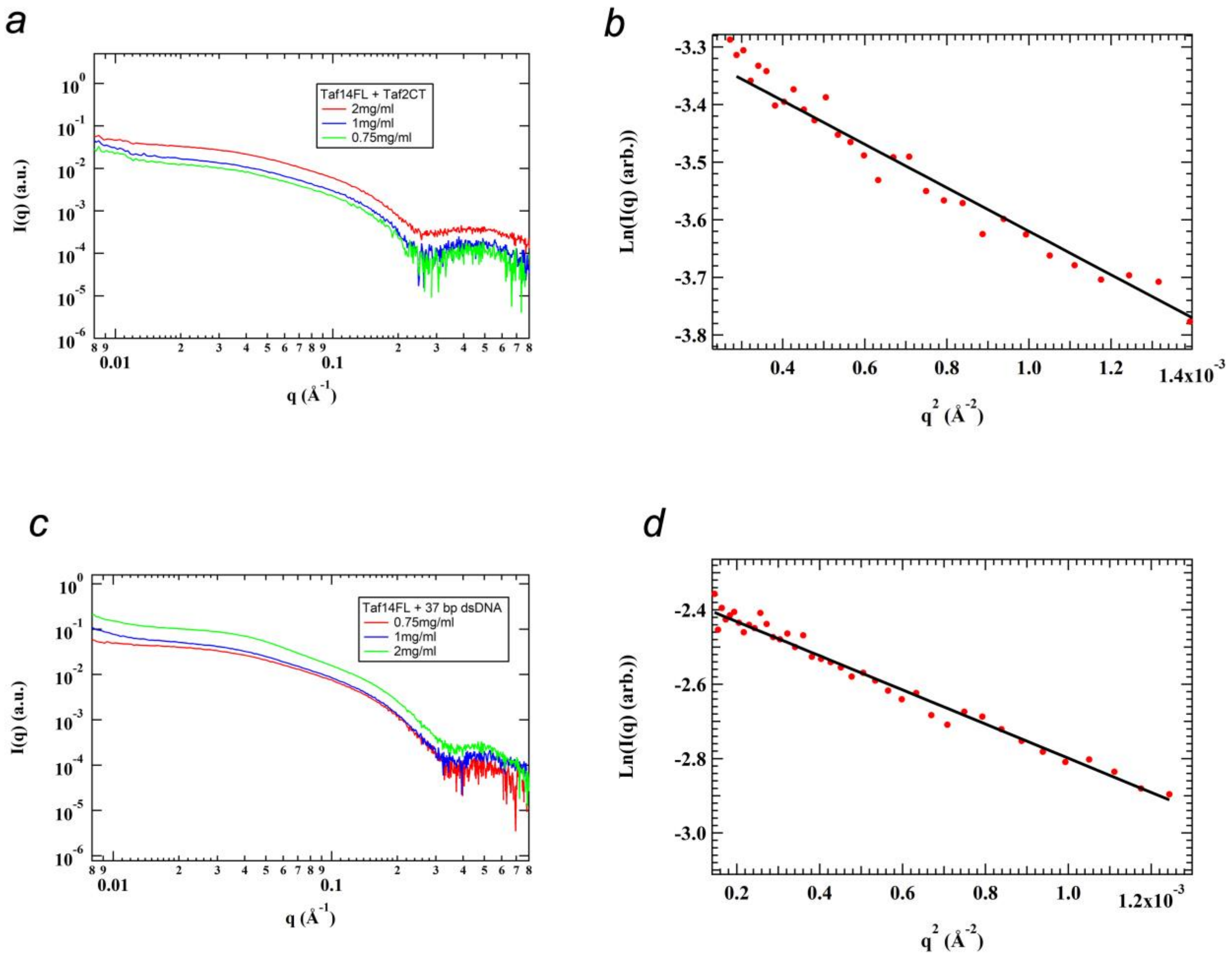

Supplementary Figure 8. SAXS profiles at function of concentrations and Guinier plots of SAXS data extrapolated to zero concentration for the $Taf14_{FL}$:$Taf2_{CT}$ complex (a, b) and the $Taf14_{FL}$:37 bp dsDNA complex (c, d). Related to Figure 3.

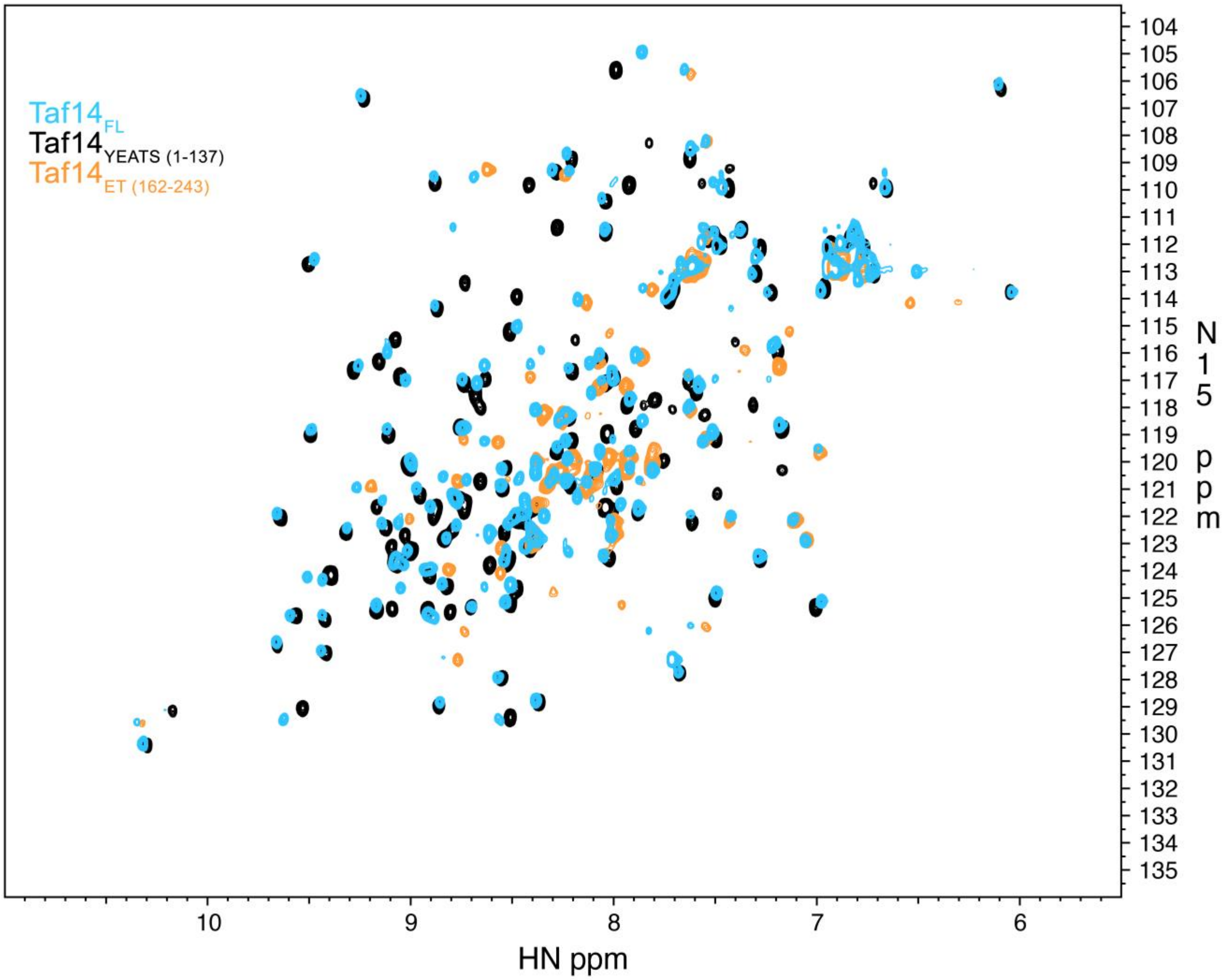

Supplementary Figure 9. The incomplete overlap between ^1H,^{15}N HSQC spectra of $Taf14_{FL}$ in the apo- state (blue) and the isolated domains: $Taf14_{YEATS}$ (black) and $Taf14_{ET}$ (orange) suggest interactions or a conformational rearrangement within full-length Taf14. Related to Figure 3.

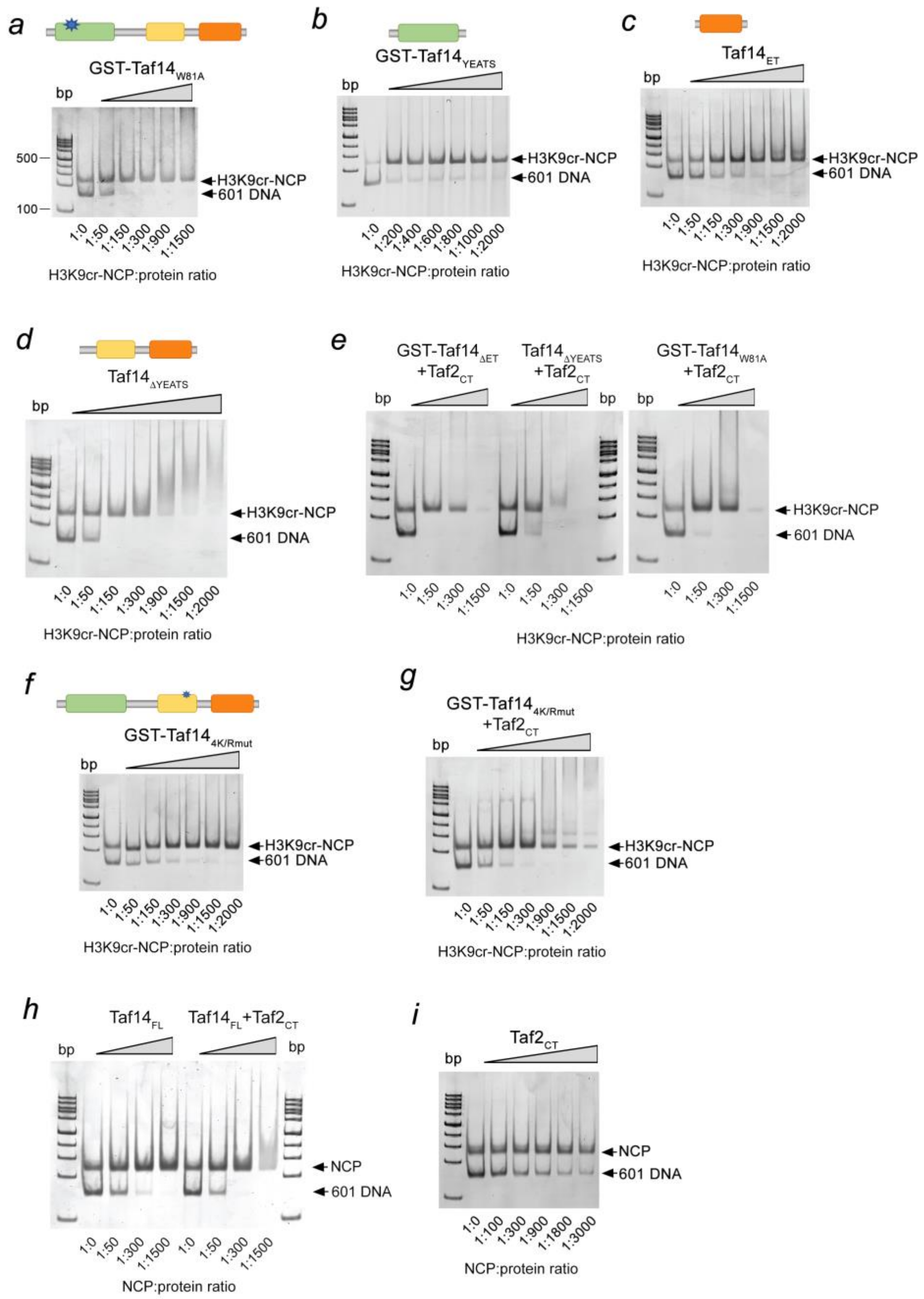

Supplementary Figure 10. Nucleosome binding activity of Taf14 and the effect of $Taf2_{CT}$**.** (a-d) EMSAs of H3K9cr-NCP in the presence of increasing amounts of indicated Taf14 proteins. Architecture of each Taf14 construct is depicted above the gels and mutations are indicated with a blue star. (e) EMSAs of H3K9cr-NCP in the presence of increasing amounts of the indicated Taf14 proteins + $Taf2_{CT}$ mixture. Taf14 and $Taf2_{CT}$ were premixed at a 1:2 ratio prior to incubation with DNA. (f, g) EMSAs with H3K9cr-NCP in the presence of increasing amount of GST-$Taf14_{4K/Rmut}$ (f) or GST-$Taf14_{4K/Rmut}$ + $Taf2_{CT}$ (g). Taf14 and $Taf2_{CT}$ were premixed at a 1:2 ratio prior to incubation with DNA. (h) EMSA with NCP in the presence of increasing amounts of $Taf14_{FL}$ (left) or $Taf14_{FL}$ + $Taf2_{CT}$ (right). Taf14 and $Taf2_{CT}$ were premixed at a 1:2 ratio prior to incubation with DNA. (i) EMSA with NCP in the presence of increasing amount of $Taf2_{CT}$. The bp marker in (b-i) as in (a). Images are from single experiment. Source data are provided in a Source Data file. Related to Figure 4.

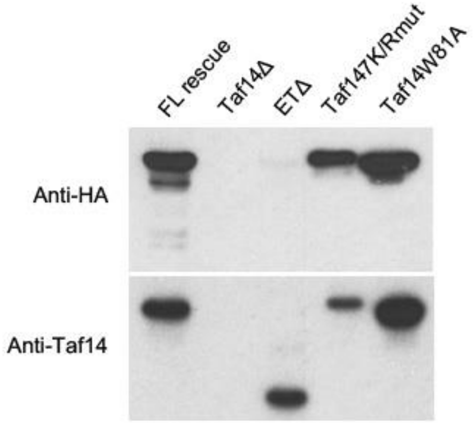

Supplementary Figure 11. Western blot analysis showing the expression of wild-type Taf14 and Taf14 mutants used for RT-qPCR, ChIP-qPCR and spotting assays. Anti-HA antibody was used to confirm sizes of wild-type and mutated Taf14. Source data are provided in a Source Data file. Related to Figure 4.

Supplementary Table 1. Data collection and refinement statistics for the structure of the $Taf14_{ET}$:$Taf2_{CT}$ complex.

	$Taf14_{ET}$:$Taf2_{CT}$
**Data collection**	
Space group	C 2 2 21
Cell dimensions	
a, *b*, *c* (Å)	47.9, 52.7, 121.5
α, β, γ (°)	90, 90, 90
Resolution (Å)	1.66 (1.69-1.66) *
R_{pim}	0.027(0.108)
$I / \sigma I$	48.5(3.6)
Completeness (%)	98.2 (78.8)
Redundancy	9.6(2.5)
**Refinement**	
Resolution (Å)	35.45-1.66
No. reflections	18094
R_{work} / R_{free}	0.1954 /0.2392
No. atoms	1443
$Taf14_{ET}$	1129
$Taf2_{CT}$	162
Water	152
B-factors	25.80
$Taf14_{ET}$	23.83
$Taf2_{CT}$	33.14
Water	32.62
R.m.s. deviations	
Bond lengths (Å)	0.006
Bond angles (°)	0.881
Ramachandran Plot	
Favored (%)	100
Allowed (%)	0
Outliers (%)	0

*Values in parentheses are for highest-resolution shell.

Supplementary Table 2. Yeast strains used in this study.

Strain	Genotype	Source
taf14Δ	*Mata his3D1 leu2D0 met15D0 ura3D0 taf14Δ::NAT*	Shanle et al., 2015
JFTAF2Δ	*Mata his3D1 leu2D0 met15D0 ura3D0 taf2Δ::hphMX4*	Feigerle and Weil, 2016

Supplementary Table 3. Plasmids used in this study.

Plasmids	Features	Source
pRS313-HA3-SSN6	CEN HIS3 p3XHA-SSN6	Michael Keogh
pRS313-Taf14(1-244)-HA3-SSN6	CEN HIS3 pTAF14-3XHA-SSN6	Shanle et al., 2015
pRS313-Taf14(1-244, W81A)-HA3-SSN6	CEN HIS3 pTAF14(W81A)-3XHA SSN6	Shanle et al., 2015
pRS313-Taf14(1-174)	CEN HIS3 pTAF14(1-174)	This study
pRS313-Taf14 (1-244, K149D/R150D/R151D/K161D/K163D/R164D/K166D) -HA3-SSN6	CEN HIS3 pTAF14(K149D/R150D/R151D/K161D/K163 D/R164D/K166D)-3XHA SSN6	This study
