# Supplementary material for: Taf2 mediates DNA binding of Taf14
Source: Nat Commun. 2022 Jun 8;13:3177. doi: 10.1038/s41467-022-30937-w (PMC9177701; doi:10.1038/s41467-022-30937-w)
Supplement: Supplementary file 3 — Description of additional Supplementary File [file 41467_2022_30937_MOESM3_ESM.pdf]

Description of additional Supplementary file

Supplementary Data 1 : Mass spectrometry analysis
