# Supplementary material for: Taf2 mediates DNA binding of Taf14
Source: Nat Commun. 2022 Jun 8;13:3177. doi: 10.1038/s41467-022-30937-w (PMC9177701; doi:10.1038/s41467-022-30937-w)
Supplement: Supplementary file 5 — Reporting Summary [file 41467_2022_30937_MOESM5_ESM.pdf]

nature portfolio

Corresponding author(s): Tatiana G Kutateladze

Last updated by author(s): Apr 19, 2022

Reporting Summary

Nature Portfolio wishes to improve the reproducibility of the work that we publish. This form provides structure for consistency and transparency in reporting. For further information on Nature Portfolio policies, see our Editorial Policies and the Editorial Policy Checklist.

Statistics

For all statistical analyses, confirm that the following items are present in the figure legend, table legend, main text, or Methods section.

n/a	Confirmed	
[ ]	[x]	The exact sample size (*n*) for each experimental group/condition, given as a discrete number and unit of measurement
[ ]	[x]	A statement on whether measurements were taken from distinct samples or whether the same sample was measured repeatedly
[ ]	[x]	The statistical test(s) used AND whether they are one- or two-sided<br>*Only common tests should be described solely by name; describe more complex techniques in the Methods section.*
[x]	[ ]	A description of all covariates tested
[x]	[ ]	A description of any assumptions or corrections, such as tests of normality and adjustment for multiple comparisons
[ ]	[x]	A full description of the statistical parameters including central tendency (e.g. means) or other basic estimates (e.g. regression coefficient) AND variation (e.g. standard deviation) or associated estimates of uncertainty (e.g. confidence intervals)
[ ]	[x]	For null hypothesis testing, the test statistic (e.g. *F*, *t*, *r*) with confidence intervals, effect sizes, degrees of freedom and *P* value noted<br>*Give P values as exact values whenever suitable.*
[x]	[ ]	For Bayesian analysis, information on the choice of priors and Markov chain Monte Carlo settings
[x]	[ ]	For hierarchical and complex designs, identification of the appropriate level for tests and full reporting of outcomes
[x]	[ ]	Estimates of effect sizes (e.g. Cohen's *d*, Pearson's *r*), indicating how they were calculated

Our web collection on statistics for biologists contains articles on many of the points above.

Software and code

Policy information about availability of computer code

Data collection	NMR experiments were carried out at 298K on Varian INOVA 600 and 900 MHz spectrometers at the UC Denver NMR Core facility. X-ray diffraction data were collected at the ALS 4.2.2 beamline, Berkeley administrated by the Molecular Biology Consortium. MST experiments were performed using a Monolith NT.115 instrument. Small angle X-ray scattering experiments were performed at beamline 12-ID-B, the Advanced Photon Sources facility (Argonne National Laboratory, Lemont, IL).
Data analysis	NMRPipe and other software listed in the Method section. Software for structure determination include HKL3000, COOT v0.9, and PHENIX v.1.18 as listed in Method section. MST software for calculation of Kd include MO Affinity Analysis as listed in Method section. The proXL conversion script (https://github.com/yeastrc/proxl-import-plink2) was used to generate mass spec analysis files.

For manuscripts utilizing custom algorithms or software that are central to the research but not yet described in published literature, software must be made available to editors and reviewers. We strongly encourage code deposition in a community repository (e.g. GitHub). See the Nature Portfolio guidelines for submitting code & software for further information.

Data

Policy information about availability of data

All manuscripts must include a data availability statement. This statement should provide the following information, where applicable:

- Accession codes, unique identifiers, or web links for publicly available datasets
- A description of any restrictions on data availability
- For clinical datasets or third party data, please ensure that the statement adheres to our policy

Coordinates and structure factors of the Taf14 ET-Taf2 CT complex have been deposited in the Protein Data Bank under the accession code 7UHE [https://www.rcsb.org/structure/7UHE]. The mass spec data have been deposited to the PRIDE database [http://www.ebi.ac.uk/pride] under accession number PXD033397.

All other relevant data supporting the key findings of this study are provided in the Supplementary Information file or from the corresponding author upon reasonable request. Source data are provided in the Source Data file.

Field-specific reporting

Please select the one below that is the best fit for your research. If you are not sure, read the appropriate sections before making your selection.

[x] Life sciences [ ] Behavioural & social sciences [ ] Ecological, evolutionary & environmental sciences

For a reference copy of the document with all sections, see nature.com/documents/nr-reporting-summary-flat.pdf

Life sciences study design

All studies must disclose on these points even when the disclosure is negative.

Sample size	present in relevant figure legends
Data exclusions	no data exclusions
Replication	present in relevant figure legends
Randomization	no randomization
Blinding	no blinding

Reporting for specific materials, systems and methods

We require information from authors about some types of materials, experimental systems and methods used in many studies. Here, indicate whether each material, system or method listed is relevant to your study. If you are not sure if a list item applies to your research, read the appropriate section before selecting a response.

Materials & experimental systems

n/a	Involved in the study	
[ ]	[x]	Antibodies
[x]	[ ]	Eukaryotic cell lines
[x]	[ ]	Palaeontology and archaeology
[x]	[ ]	Animals and other organisms
[x]	[ ]	Human research participants
[x]	[ ]	Clinical data
[x]	[ ]	Dual use research of concern

Methods

n/a	Involved in the study	
[x]	[ ]	ChIP-seq
[x]	[ ]	Flow cytometry
[x]	[ ]	MRI-based neuroimaging

Antibodies

Antibodies used	Antibodies used for assays were as follows: anti-HA blot 3F10 (Sigma-Millipore, cat # 12013819001, 1:2000 dilution), anti-HA IP 12CA5 (made in house, 2.5 ug per reaction), anti-Taf3 (made in house, 1:1000 dilution), anti-Taf7 (made in house, 1:2000 dilution), anti-Taf8 (made in house, 1:1000 dilution), anti-Taf14 (made in house, 1:2000 dilution), anti-rabbit Fc HRP-conjugated secondary antibodies (Abcam, 1:10,000 dilution), anti-HA (Bethyl, cat # A190-108A, 1:1000 dilution)
Validation	All antibodies validation are available on the manufacturers' websites.
